# Evaluation of Sustained Acoustic Medicine for Treating Musculoskeletal Injuries in Military and Sports Medicine

**DOI:** 10.2174/18743250-v16-e221130-2022-8

**Published:** 2022-12-28

**Authors:** Rod Walters, John Kasik, Cassie Ettel, Ralph Ortiz

**Affiliations:** 1 NATA Hall of Fame, Walters Inc. Consultants in Sports Medicine, Columbia, SC, USA; 2 Atheltic Training and Sports Medicine, University of South Carolina, Columbia, SC, USA; 3 Atheltic Training, Jacksonville Jaguars, Jacksonville, FL, USA; 4 Cayuga Medical Center, Medical Pain Consultants, Dryden, NY, USA

**Keywords:** SAM, NCAA, Acoustic medicine, PRP, Injuries, Recovery

## Abstract

**Background:**

Musculoskeletal injuries are common in collegiate, professional, and military personnel and require expedited recovery to reduce lost work time. Sustained acoustic medicine (SAM) provides continuous long-duration ultrasound at 3MHz and 132mW/cm^2^. The treatment is frequently prescribed to treat acute and chronic soft tissue injuries and reduce pain. The objective of this study was to evaluate the efficacy of SAM treatment for musculoskeletal injuries and accelerated recovery.

**Methods:**

An 18-question electronic survey and panel discussion were conducted on Athletic Trainers (ATs) using SAM treatment in professional, collegiate, and military sports medicine. The survey included both qualitative and quantitative questions. In addition, a panel discussion discussed SAM effectiveness with expert ATs. Power calculation of sampling and statistical evaluation of data was utilized to generalize the results.

**Results:**

Survey respondents (n=97) and panelists (n=142) included ATs from all National Athletic Trainers Association districts. SAM was primarily used for musculoskeletal injuries (83.9%, p<0.001) with a focus on healing tendons and ligaments (87.3%, p<0.001). SAM treatment was also used on joints (44.8%), large muscle groups (43.7%), and bone (41.4%). SAM provided clinical improvement in under 2 weeks (68.9%, p<0.001) and a 50% reduction in pain medication (63%, p<0.001). In addition, patients were highly receptive to treatment (87.3%, p<0.001), and ATs had a high level of confidence for improved function and returned to work after 30-days of SAM use (81.2%, p<0.001).

**Conclusion:**

SAM is an effective, safe, easy-to-use, noninvasive, comfortable, and versatile therapeutic for healing musculoskeletal injuries.

## 1. BACKGROUND

Professional and college sports are important industries in the United States. By 2025, it is estimated that the professional sports market will be worth $83 billion [1]. College sports are also financially vital for their schools and communities. The total athletics revenue reported among all National Collegiate Athletic Association (NCAA) athletics departments in 2019 was $18.9 billion. By 2025, the NCAA is expected to generate $990 million from television rights agreements alone [2]. Players must stay in the game and remain healthy; however, high-level sports are physically demanding to train for and play [3 – 9]. Physical conditioning is rigorous and extends from the preseason through the postseason [10 – 12].

Soft tissue injuries and recovery are affected by the nature and location of the trauma and the player’s physical, nutritional, and emotional condition. Many treatment options exist for strains and injuries [13 – 18]. The most basic treatments are RICES (Rest, Ice, Compression, Elevation, Stabilization), massage, and oral analgesics for pain and inflammation. Therapy choices once only included EMS (electrical muscle stimulation), TENS (transcutaneous electrical stimulation), laser therapy, therapeutic ultrasound, shockwave therapy, stretching exercises, and in some cases, PRP (platelet-rich plasma) injections and biologics [19 – 29]. These treatment options are limited to the relatively short time-course of treatment and subtle biophysical impact on tissue regeneration [30 – 32].

One of the most common therapeutics for soft tissue pain is non-steroidal anti-inflammatory drugs (NSAIDs) [33]. They reduce inflammation and pain by regulating the activity of cyclooxygenase enzymes COX-1 and COX-2 [34 – 36]. They are available both in oral and topical forms. The extended use of oral NSAIDs such as paracetamol and celecoxib can lead to adverse effects on multiple organs; topical applications such as diclofenac, while reducing NSAID adverse systemic effects, show reduced efficacy due to limited penetration through the skin [37 – 41]. Therefore, an effective non-invasive approach is required to reduce the adverse effects of oral NSAIDs and increase the bioavailability of localized topical NSAIDs to improve the efficacy of musculoskeletal pain reduction.

In the context of sports medicine, Sustained Acoustic Medicine (SAM) therapy provides continuous high-frequency, low-intensity long-duration ultrasound of 3MHz, 132mW/cm^2^, and 18,720 Joules of energy to a specific injury site. Multiple clinical and animal studies have demonstrated the ability of SAM to enhance soft tissue regeneration and healing by increasing tissue temperature and circulation, reducing inflammation, and relieving pain in acute and chronic injuries [42 – 46]. Due to the efficacy profile, and non-narcotic and non-surgical nature of the treatment, the application of SAM has gained widespread use among Athletic Trainers (ATs) in the United States. As a result, it has rapidly become a preferred method to accelerate athletes’ healing to return them confidently to play in a highly competitive environment.

SAM is considered an effective therapy for professional athletic trainers [47]. Previously, professional sports ATs reported their treatment preferences, satisfaction, impact on return to sports, and the decision-making process in injury treatment with SAM. The open panel discussion among the professional sports ATs studied the application of SAM treatment and the treatment effects on their athletes [47]. This study includes broader participation from members of the National Athletic Trainers Association (NATA) across the United States who regularly use SAM. The NATA is a membership organization of all ATs licensed, certified, or registered in all states except California. NATA includes a broad group of United States Military, sports medicine, industrial, rehabilitation practices, doctors’ offices, and athletic training facilities and has 45,000 members worldwide [48]. The main theme of the survey and panel discussion was assessing SAM’s role in the rehabilitation, return to work and pain management. The theme was selected using previous observational and clinical studies published in the literature.

## 2. MATERIALS AND METHODS

The study consisted of independent survey analysis and discussion by a panel of ATs from colleges, professional sports, and the U.S. military. This study aimed to determine the efficacy of SAM with diclofenac gel in healing trauma from physically rigorous training and sports using real-world outcomes in treating injuries. Institutional review board (IRB) certification was obtained from Advarra IRB #00000971 for the study.

### 2.1. Participant Selection

Professional and college sport ATs and military ATs routinely use SAM. We used an email survey of these athletic trainers and a follow-up live panel discussion to determine their use and experience with SAM to treat injuries. Survey and panel participation was voluntary and did not include costs or fees.

The 18-question survey was emailed to members of the NATA using SurveyMonkey^®^ 7-days before the panel review. Survey participants were requested to have experience using SAM treatment for sports-medicine-related injuries and participate in live and virtual panel discussions in reviewing real-time results at the time of the meeting. The data was collected by Survey-Monkey^®^ cloud-based data capture.

### 2.2. Data Collection

The acquisition of data on the practical application of SAM was two-fold. First, ATs were asked to complete questionnaires with specific questions about SAM treatment, clinical utilization, use of topical therapeutics, and overall confidence in the therapeutic approach. This included questions about patient attitude, treatment response, and compliance (Table 1); data were collected and coded using Survey-Monkey ^®^. A total of 97 written responses were received over seven days.

In the second phase, an open panel review of survey results and questions was conducted via live meeting and Zoom video conferencing. 142 ATs attended the 2-hour long virtual-and-in-person panel meeting. The panel was open to everyone in the panel to comment, provide best practice feedback, and review the survey results presented by the hosts. There was no external observer in the panel. Eighteen participants spoke for the direct review of questions. The discussion panel leaders with prior experience in SAM treatment included the paper’s authors, Dr. Walters, ATC, EdD, and Dr. Ortiz, D.O., MPH-certified sports physicians and athletic trainers. Both interviewers are males, Dr. Walters has 27 years of experience in physician education, sports medicine, and training, and Dr. Ortiz has 25 years of experience as a researcher and a Family medicine practitioner with specialties in pain management and sports medicine. The participants were selected based on their experiences being athletic trainers and physical therapist and their years of experience with patients. The interviewers had no relationship with the participants. The participants were informed about the interviewers’ credentials and background, the interview’s purpose, and the survey’s synthesis. The panel discussion was closed after the completion of the panel session, and no-repeat interviews or panel discussions were conducted. The data was collected using the standard USA health system requirements and recorded for analysis. Data was analyzed in its raw form without any saturation. The guided discussion focused on specific examples and used case treatment scenarios representative of the survey results. The authors’ synthesis of the discussion and summary of the panel review covered relevant aspects of collegiate sports athletic training, professional sports athletic training, military training, pain management, physical medicine, and rehabilitation. The data was analyzed using content analysis based on the responses from the participants. Field notes were made during the panel discussion and recorded for data analysis. The data was not saturated, considering this was a first-time study.

### 2.3. Statistical Analysis

According to the U.S. Bureau of Labor Statistics, approximately 32,100 ATs were employed in 2019. The NATA database further breaks down the employment of ATs, which identifies areas of ATs employed actively: 6,099 or 19% College/University, 642 or 2% Professional Sports, and 642 or 2% Emerging Settings, including the military [48]. Therefore, the survey population was estimated to be 7,383 for collegiate, professional, and military ATs in the United States. A sample size power analysis for the survey with 95% confidence and 10% margin of error provides the sample respondence size of n=95. All the statistical analyses were conducted using R (The R Project for Statistical Computing). Results from the survey data were expressed in percentages and analyzed with a standard two-sample proportion test, t-tests, and ANOVA as appropriate for response grouping or individual response selection with significance set at p<0.05. Power calculation of sampling was utilized for generalizability of respondents surveyed, and statistical evaluation of survey data was used to determine if trends in the survey data and subgroups were meaningful among the ATs in using SAM therapy.

## 3. RESULTS

### 3.1. Survey

Data from the written survey and live discussion of athletic trainers are presented here. The respondents provided information on their experience with SAM. They included their positions and levels of experience as ATs, the number of patients treated with SAM, and their age range. Members of all ten NATA districts in the United States participated in the survey (Table 2). Responses for the clinical utilization of SAM included the type of injuries treated, length and duration of treatment, and sonophoresis of topical NSAIDs for enhanced pain relief. They also gave their input on the acceptance and compliance of the athletes using SAM, including patient emotional state, expectations, and satisfaction with treatment.

#### 3.1.1. Respondents

Most respondents were ATs in college athletics programs (80.4%). Professional sports ATs (15.2%), military (3.3%), and private sector healthcare professionals (1.1%) rounded out the survey population. The group had 16.5 ± 9.7 years of experience in the healthcare field. Over 77.0% of the survey respondents had used SAM for over one year, while the average use of SAM treatment was 3.4 years. Most participants reported their patients’ age range from the late teens to early twenties. A small number of ATs reported patients up to their mid-thirties in age. On average, the total number of patients ATs treated with SAM was 29.8 ± 55.9.

#### 3.1.2. Application Sites and Use of SAM Treatment

The primary reason for SAM treatment was to heal specific injuries (83.9% of respondents, p<0.001). Other uses were pain reduction (39.1%), general recovery (10.3%), and post-operative healing and pain management (8.1%). Most survey participants (87.4%, p<0.001) utilized SAM on injuries to tendons and ligaments. SAM was also frequently employed to heal injuries of the joints (44.8%), large muscle groups (43.7%), and bone (41.4%).

#### 3.1.3. Timing and Length of Treatment for the Healing Response

SAM was prescribed for 2-4 hours per day by a significant majority of the surveyed ATs (60.9%, p<0.001). Some replied that their injured athletes used it for more than 4 hours daily (24.2%). A minority of ATs recommended it for only 1-2 hours daily (14.9%).

Some ATs reported that their athletes responded to SAM within one week (25.3%). However, most gave 1-2 weeks as the usual length of treatment needed to see a definitive healing boost from SAM (69%, *p*<0.001). Virtually all the remaining responses indicated improvement by 4-8 weeks (5.8%). Eighty-one-point four percent (81.4%) of all respondents gave an 80-100% confidence expectancy that SAM would provide a positive tissue healing response within 30 days (*p*<0.001).

#### 3.1.4. Pain Relief

**Fig. (1).**
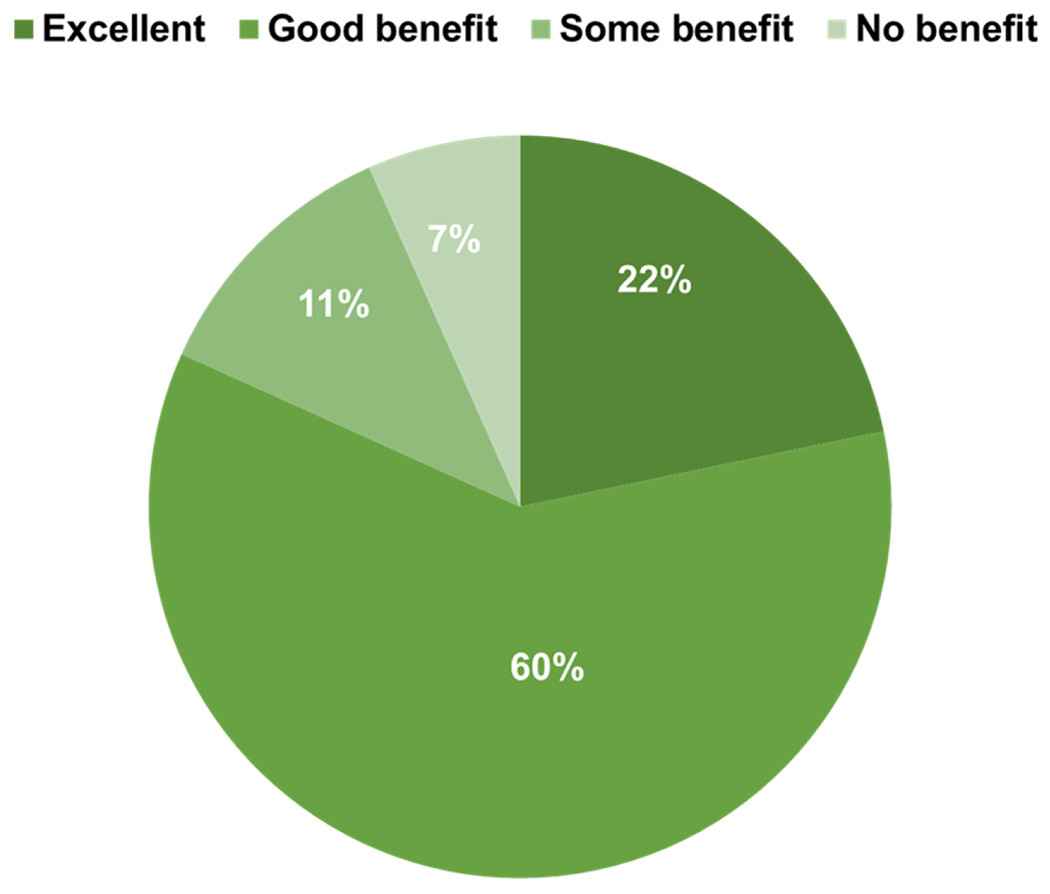
Patient benefits from the use of SAM combined with topical medication for pain management.

SAM can be used as a stand-alone treatment to alleviate pain or in conjunction with topical non-steroidal anti-inflammatory drugs (NSAID) such as diclofenac and dexamethasone. Research shows that SAM enables the topical drug to penetrate more deeply through the skin and into the injured tissue, providing localized pain relief [49, 50]. However, survey replies showed that less than half of ATs (46%) used topical NSAID with SAM at least 25-75% of the time within the past 3 months, whereas the rest (54%) used it less than 25% of the time with no significant differences between responses. Even so, pain reduction was a widely reported benefit, and a significant 82.6% majority of survey respondents agreed that at least half of their patients used less medication while on SAM therapy (Table 3). Finally, 69% of the surveyed ATs have some form of experience using SAM combined with topical NSAID for treatment, and of this subgroup, the majority agreed that multi-hour SAM-delivered sonophoresis provided a Good to Excellent benefit in pain management for patients (82%, p<0.001) (Fig. 1).

#### 3.1.5. Patients’ Experience

Seventy-two-point nine percent (72.9%, *p*<0.001) of ATs reported that their injured athletes showed a major mood change or depression due to their injury. Thus it is not surprising that most injured athletes were either completely receptive (42.8%) or mostly receptive (44.8%) to using SAM to enable and accelerate their recovery (87.6%, *p*<0.001). When the athlete was permitted to resume playing, 32.6% were positively surprised by the healing outcome, 38.4% felt the injury healed faster than expected, and 16.3% felt that the treated area was strengthened more than before. SAM provides a clear boost to the attitude and morale of injured athletes by enabling them to heal quickly, regain strength in the injured area, and feel confident in resuming their sport (Table 3).

Health insurance coverage for SAM was required or very important according to 58% of the ATs, while 42% called it slightly important or unimportant, having no significant difference between groups.

### 3.2. Panel Discussion

Two (2) physicians led the panel discussion with in-person and virtual attendees. Participants identified themselves as licensed or certified ATs, physical therapists, and medical professionals involved in professional sports, college sports, and military sports medicine. The format was an open question-and-answer forum, where the panel moderators reviewed the survey results and introduced questions based on the survey. Participants responded with their own experiences in each subject area.

**Question:** *When and how soon do you notice the benefits of SAM on patients?*

**Response:** SAM treatment provides a strong clinical response in 1-2 weeks. There is an immediately improved joint range of motion, vasodilation and heating of tissue in a single treatment. Adding a topical NSAID in the coupling patch and SAM treatment can provide near-immediate relief from pain for patients using SAM.

**Question:** *Is there any treatment difference based on the size (BMI) of the patient as it relates to the efficacy profile for osteoarthritis* [51]?

**Response:** SAM is an ultrasound-based therapeutic and will penetrate deep tissue. An osteoarthritis study was performed with patients with high BMIs treating relatively larger joints, which found SAM effective [42, 51, 52]. Ultrasound wave interactions allow two transducers combined to produce a higher cumulative treatment depth, allowing the ultrasound to penetrate deeper into the tissue. Therefore, for a larger patient, two transducers applied close together will have an additive effect for a therapeutic benefit. Other types of energy therapy (electromagnetic), for example, laser therapy, are not additive like mechanical/ultrasonic energy in this way and cannot effectively treat a larger joint the way SAM can.

**Question:** *How soon can you apply SAM after an acute injury when treating chronic vs. acute injuries? How does treatment benefit an acute injury?*

**Response:** It is more beneficial to treat an acute injury rather than resolve a lingering injury later. It is unnecessary to let the tissue initiate the healing process before applying SAM; the sooner treatment is applied, the better it is for returning the athlete to play. SAM stimulates all phases of the healing process, which has been documented in science. With an injury such as a rotator cuff tear that requires surgical intervention, SAM can be used immediately post-operatively; however, it is not a sterile device and cannot be put over fresh wounds, so the device must be applied proximal to the incision sites. In this scenario, ATs use SAM as soon as the patient can tolerate the treatment on their skin.

**Question:** *Where do ATs use SAM on the body most often?*

**Response:** SAM is used by most participants for tendon and ligament injuries. Treatment sites include shoulder and bicep tendonitis. Patellar tendonitis overuse injuries from basketball jumping respond well to SAM. Patients are told what to expect and use the device for 4-hour daily treatments. Patients show good compliance and recover within a couple of weeks. Football ATs like SAM for their tendon and hamstring injuries. We use the treatment regularly during our 3-hour team meeting windows.

SAM is beneficial in healing bone fractures. The panel discussed the treatment of athletes who have fractured metatarsals, clavicles, and ribs. After 3 weeks of SAM treatment, fractures are usually halfway healed, and at 6 weeks, they are healed completely, allowing the athlete to play again. The panel agreed that it takes six to eight weeks for a minor fracture to heal completely with SAM.

**Question:** *How long does it take for your patients to respond to SAM treatment?*

**Response:** After 1-2 weeks, the treatment benefit is very clear, with treated patients showing a better range of motion, improved function, and about 50% faster healing than patients not receiving SAM treatment.

**Question:** *What conditions show improvement in response in less than a week?*

**Response:** About a quarter of the respondents said their patients responded to SAM in less than a week. In less than one week, conditions improved, including acute tendonitis, muscle damage, inflammation, joint stiffness, and severe metatarsal pain.

**Question:** *How quickly do patients respond to SAM with and without topical NSAID?*

**Response:** Diclofenac within the SAM patch is helpful in older populations, usually alleviating pain within a day or two. A football athlete with a rib fracture showed a significant decrease in pain within 3 weeks. The patient was not x-rayed weekly to minimize radiation exposure. After 5 weeks of SAM therapy, 7 days a week, 4 hours a day, he healed without tenderness and could play.

**Question:** *About half of survey respondents did not use topical drugs with SAM. What limits or prevents you from using it? When would you think about trying it for a patient’s treatment?*

**Response:** SAM alone works well for pain modulation by helping soft tissue injuries heal. ATs would consider using a topical drug with SAM treatment if the patient does not respond to SAM alone.

**Question:** *What are the most common injuries you target for healing with SAM?*

**Response:** In football, soft tissue traumas are treated, including hamstring strains, ankle sprains, and tendinopathies. Both acute and chronic injuries respond well to SAM.

**Question:** *Is there anything in the patient demographic where you only treat pain with SAM? Is reducing pain a secondary healing effect, or is it something you’re targeting?*

**Response:** Chronic joint pain, where athletes can’t pinpoint the source of their pain or an arthritic shoulder or knee that may be already past the healing stage, are targets for SAM. Good results are seen when treating just for pain.

**Question:** *The military will be the second-largest employer of ATs in the United States. Are you treating acute trauma like athletes or more chronic conditions?*

**Response:** It is a combination of both acute and chronic injuries. Military personnel is “tactical athletes.” The major type of injury varies with the location of deployment. There is no “off-season,” as there is for athletics, for recovery. The training cycle is constant, and they need to recover. The military works with the “aging operator” population 28-40 years old with chronic arthritic joints. It is high mileage, high wear-and-tear job. Personnel sustains a lot of acute injuries during training.

**Question:** *Are military patients faced with cognitive dysfunctions due to medications required to ease the pain?*

**Response:** Military patients are not significantly challenged with pain drugs that change their mental acuity or ability to perform during training. There is mostly no pharmacological intervention because patients don’t want to become compromised and unable to fight. Ex-Military pain patients are good patients for SAM. They are compliant with their treatment regimen. Providers are encouraged to focus on SAM in aging populations with chronic injuries, not just younger athletes.

**Question:** *About 60% of respondents dosed their patients with SAM for 2-4 hours a day. When would you recommend more than 4 hours?*

**Response:** It is time-dependent and depends on our time with the athlete. Most patients take SAM home for a 4-hour dose. If that is not feasible, then a one to two-hour dose during meetings. Some athletes are nervous that they will have an adverse reaction to SAM, so we set the first dose for two hours. We have to tell patients to wear it a little longer than 4 hours to sustain healing and to bump it up to 20,000 joules for patients who can only wear it 2-3 times per week. It’s tough sometimes to get the SAM treatment time in. Travel, hotel time, and downtime are good for getting in multi-hour treatments on athletes.

**Question:** *How receptive are your patients to treatment and following instructions? How are you coaching patients to ensure they are using SAM appropriately?*

**Response:** First, pick patients who will not lose the device and use it. It is also essential to educate the patient on how the device operates. For example, sometimes it vibrates and turns red. Tell them, “Do not take it off!” Some patients take it off too early because they misinterpret its signals, especially on their feet, so make sure they understand what will happen when used.

**Question:** *How do you coach patients to ensure they use it appropriately?*

**Response:** Do not throw it away! Do not take it off if it vibrates and the light turns red and blue. We tell the athletes to stay with treatment and remove the device if it hurts.

**Question:** *How has treatment reduced the need for oral pain medicine?*

**Response:** More than 80% of respondents say at least 50% of their athletes take less oral pain medication, and 20% report a substantial reduction in medication with SAM treatment. Patients can use a local analgesic patch with SAM to load the area with NSAID without systemic medication. It is a great approach to reducing total NSAID use on the body.

**Question:** What are the early indications for treatment success?

**Response:** I think the biggest indication is that athletes ask for it because it works for them.

**Question:** *Rate your satisfaction with drug delivery and sonophoresis with SAM.*

**Response:** 55% found that SAM with a topical agent is helpful, but 35% don’t use a topical agent at all. If an athlete is nonresponsive to pain reduction with SAM or has an inflammatory healing response, the addition of a topical agent is recommended.

**Question:** *About 73% of respondents stated that injured athletes showed noticeable major mood changes after an injury. Are there certain stigmas out there that, once damaged, a player can no longer play?*

**Response:** Depression directly correlates to the amount of time the athlete is expected to miss across most sports settings. They will be more depressed if they are out longer. An athlete with a sprained ankle could be out for 6 weeks. There’s a negative stigma around being pulled out of the sport with an injury or illness. If a college player is hurt in their draft-eligible year, it is important to understand what is at stake situationally.

**Question:** *The survey shows a 50% increase in confidence levels in athletes returning to play after SAM treatment. Is this confidence important?*

**Response:** SAM is an important and advanced treatment option for musculoskeletal injuries. For athletes, SAM noticeably augments their rehabilitation and return to sport. We ensure the athletes are reacclimatized to 100% effort during the return to play and utilize SAM during reintroduction. A multimodal approach is recommended and helps rebuild the athletes’ confidence before returning.

**Question:** *Where would you like to see more research and evidence on sustained acoustic medicine as healthcare providers? Do you want to see more basic mechanisms of action on biological response? More clinical trials to help understand its effect on athletes? What area of research is most influential to you?*

**Response:** Human clinical outcomes, with possible effects on ligament tears that don’t involve surgery, would be better so that athletes are not out for as long.

**Question:** *What would you like to see as a control group in clinical studies?*

**Response:** Standard of care compared to standard of care plus SAM would compare with what is usually done and look at the factors involved. Another interesting study would be using mechanical stimulation with SAM to stimulate full-body recovery instead of just the area of injury. Does wearing the SAM unit help recovery even after the athlete returns to active play?

ATs are interested in seeing if SAM could help with nerve injury, pain in the lower back, or arthritis.

### 3.3. Summary of Q&A Session

The panel of ATs agreed that SAM is a beneficial therapy for athletic injuries, including trauma to joints, muscles, bones, tendons, and ligaments. SAM is also part of their treatment protocol for pain management, general recovery, and post-operative healing. Most ATs prescribed SAM treatment for 2-4 hours per day. In addition, participants felt that SAM should be utilized as soon as possible after an injury, the exception being that it should not be applied over an open wound.

Sonophoresis of topical pain medications with SAM was used by less than half of the ATs; nevertheless, 83% indicated that SAM alleviated pain in their patients.

An important observation is that SAM therapy gave ATs and athletes high confidence in their recovery. In addition, enhanced speed and strength of the recovery and a reduction in the use of oral pain medications led to positive physical and psychological responses. The participants were asked to provide their feedback about the panel session toward the end of the panel. Participants were excited about the panel session and encouraged to hold such sessions in the future.

In the future, panel participants would like to see data on the non-surgical healing of ligament tears and general use to stimulate full-body recovery.

## 4. DISCUSSION

In the last decade, SAM has been introduced to the healthcare system as a non-invasive, versatile, and effective treatment that promotes the rapid healing of sports injuries. It specifically targets the injury site, avoiding systemic adverse effects while enhancing tissue regeneration over multiple hours of treatment. Pain reduction from healing is another benefit, magnified with the addition of topical NSAIDs to SAM therapy. Topical medications are proven to absorb into the tissue faster and deeper when used with SAM [49, 50, 53]. The application of the SAM provides continuous ultrasound pulse-induced mechanical force, and microbubble cavitation and heat increase the skin’s permeability, the limiting barrier for the topical NSAIDs, increasing the bioavailability of the diclofenac at the injury site [46, 54 – 58].

Therapeutic ultrasound has been manually administered for decades by ATs for short 5-10 minute durations throughout injury rehabilitation. The manual process of traditional therapeutic ultrasound treatment poses challenges for daily therapy administration. The SAM therapeutic approach is relatively new to healthcare but advancing rapidly across sports medicine with wearable, autonomous, self-administered long duration therapeutic ultrasound. While the ultrasound frequency, power output and waveform are similar between SAM and traditional therapeutic ultrasound, the long-duration daily application of SAM allows for substantially more ultrasonic stimulation of tissue (18,720 Joules *vs*. <2000 Joules) to rapidly accelerate tissue healing every day. The Food and Drug Administration (FDA) provided 501(k) clearance for SAM in 2013, acknowledging that it is safe and effective (59). The treatment applies continuous high-frequency ultrasound energy (100% duty cycle) at 3 MHz, 0.132W/cm^2^, 1.3W, with a total energy of 18,720J. This generates and sustains significant tissue heating up to 14°C on the skin surface and 4°C in deep tissue [59]. The recommended delivery duration is between 1 and 4 hours based on clinical dosing protocols guided by randomized controlled trials. Multiple studies demonstrate the effectiveness of SAM in relieving pain from osteoarthritis, myofascial pain, muscle spasms, and healing and improved functionality after various soft-tissue tissue injuries [42, 43, 45, 46].

Several different but comparable methods measured function, health improvement, and pain assessments in SAM clinical trials. The VAS, or Visual Analog Scale, asks the patient to plot their relative pain sensation on a linear or analog scale [60, 61]. It is similar to the NRS, Numerical Rating Scale, where patients circle a number from 1-10, or 1-100, to rate their pain level [62, 63]. The Global Rate of Change (GROC) scale is a patient self-assessment that determines an intervention’s effect by quantifying a patient’s improvement or deterioration over time [62]. The WOMAC (Western Ontario and McMaster Universities Arthritis Index) is a more detailed questionnaire that quantifies patient pain, stiffness, and physical function [64, 65]. These ratings have been applied in SAM studies and demonstrate that SAM consistently alleviates pain, improves function, and improves patient treatment satisfaction in various musculoskeletal conditions.

VAS scores showed 52% improvement after rotator cuff injury (n=4), a 50% reduction in chronic tendon pain (n=25), and a 40% decrease in pain for osteoarthritis (n=47) after 6 weeks of SAM therapy [66 – 68]. Trapezium muscle spasm was reduced by 25% (*p*<0.05) in a clinical case series of 30 athletes [44]. Chronic myofascial pain sufferers gained up to a 1-point increase in GROC score and improved by 25% after SAM therapy [69]. Pain resulting from tendinopathy was significantly alleviated in a 20-patient study by Best *et al*. 2015 [70]. Handgrip strength in these subjects increased by 2.83 kg (p=0.02) after a 6-week course of SAM therapy [70]. Patients in a 6-week study on joint pain displayed a 1.96 point (*p*<0.001) improvement on the NRS and 505 points (*p*=0.02) on the WOMAC index. After only 4 weeks of SAM treatment in a double-blind placebo-controlled clinical trial, treated subjects showed a 2.61-point reduction in pain on the NRS scale (*p*<0.001) compared to placebo and a 2.84-point improvement in GROC score compared to a 0.46 point improvement in the placebo group (*p*<0.001) [71]. The versatility of SAM allows it to be used as an add-on therapy with other therapeutic modalities. Case reports by Draper *et al.* 2020 showed a significant average improvement of 3.33 +/−0.82 on the NRS pain scale and a 100% satisfaction rating on device usability when SAM was combined with traditional therapy for injured athletes [72].

The studies in the literature consistently corroborated the effectiveness of SAM in healing musculoskeletal pathologies. Steady, continuous ultrasound therapy increases healing by increasing temperature and vasodilation by amplifying cellular and molecular pathways, hastening tissue debris removal, and regenerating new matrices [19]. When administering SAM immediately following a fresh injury, caution is warranted for the rapid vasodilatory effects and increased blood flow into the injury site. Basic treatments such as RICES, massage, and oral analgesics are now being used in addition to SAM. Other technologies, such as EMS (electrical muscle stimulation), TENS (transcutaneous electrical stimulation), laser therapy, and PRP (platelet-rich plasma) injections, have limited benefits [19 – 23, 73]. Additionally, they cannot be self-administered and require the patient to attend scheduled therapy sessions to receive treatment. PRP injections, or platelet-rich plasma injections, use the patient’s platelets to enhance bone and tendon trauma healing. While promising, PRP is invasive and time-consuming. PRP requires the injured person to undergo a series of blood withdrawals, platelet enrichment of the blood samples, and reinjection of the fractionated plasma into the injury site [22, 74, 75].

In contrast, SAM is a fast, simple topical application yielding rapid healing without causing additional trauma to suffering athletes. Additionally, SAM may be used for multiple hours daily to actively promote the body’s healing. The contraindications for SAM mirror traditional therapeutic ultrasound and include restrictions of use over an open wound, an active malignancy and on the reproductive organs, cranium and abdominal area of a pregnant woman. Most patients can tolerate SAM well; however, patients who have skin sensitivity to heat or cannot apply SAM treatment daily may not be appropriate for treatment. Overall, Sustained Acoustic Medicine has unique advantages over all other therapy technologies and has shifted the care paradigm for soft-tissue injuries for sports medicine providers.

Pain reduction, expedited recovery, and improved emotional well-being for the athletes using SAM were clearly substantiated in this present study. The increased role of SAM as an adjunctive therapy to treat athletic injuries is highlighted here, supporting a recent review by Draper and Best [47]. While following the format of their original review, this expanded data set broadens the understanding of the real-world use of SAM in athletes under extreme physical demands. Candid input from professional athletic and military ATs shows a consensus that SAM is a superior healing technology. These results are generalizable across these three healthcare professional groups with 95% confidence based on survey power and statistical analysis.

The survey and discussion panel confirmed that their primary use of SAM was to heal specific injuries and reduce pain, either with or without additional topical pain relief medication. The use of daily SAM treatment on musculoskeletal injuries by ATs was for curative treatment, and there was a clear indication for SAM in treating chronic injury symptoms such as osteoarthritis and muscle spasms. This novel finding of SAM use is similar to how ultrasound-based bone growth stimulators are used to heal fractures in the home setting, however dissimilar to traditional therapeutic ultrasound, which is generally utilized for short-term palliative care rather than curative treatment.

Most ATs prescribed the SAM device for the recommended time of at least 2-4 hours per day. The ATs reported that they and their athletes felt confident in SAM treatment, and they reported satisfaction in their healing with this approach. Patient compliance was good. The key point was that these athletes could use SAM daily, even on team travel days, because they were not tied to physical therapy appointments or sitting at a table receiving a passive therapeutic modality. In addition, SAM is portable and self-administered, enabling the patients to keep it with them and use it as often as prescribed.

The ATs agreed that the application of SAM should begin as quickly as possible after an injury occurs. They found it useful at any stage of healing acute and chronic injuries. They shared individual anecdotes of rapid healing and pain reduction in difficult sites. Most importantly, the majority felt that their athletes recovered their confidence and strength. Confidence is an indisputably critical factor for athletes who push themselves to their physical limits in front of huge crowds of spectators. Confidence is even more important for military operators whose lives depend on strength and agility.

Limiting factors for SAM therapy were minor. The discussion agreed that patients using the device unsupervised should be informed of what to expect from the device. Patients with high BMI can “double up” on transducer placement to maximize ultrasound penetration at that site. Insurance coverage of SAM therapy was important to most SAM users. Medical insurance companies require a preponderance of evidence from comparable studies to convert their acceptance of SAM from an “investigational” device to a ” medically necessary device.” Evidence and healthcare provider advocacy for the benefits of SAM therapy should continue to be expanded to facilitate broader and more readily available insurance coverage for this effective treatment option.

## CONCLUSION

College and professional sports have a significant impact on the United States economy. In addition, the United States Military employs millions of Americans. Military, professional, and college athletes undergo demanding physical training. Participation in their sport or mission depends on physical strength and agility. When injuries occur, they are treated immediately with the best FDA-approved technologies available. One of the favored methods is SAM.

SAM is an effective non-invasive healing tool for acute and chronic connective tissue and muscle injuries. It provides deep pain relief without the use of oral medications. Because it is specific to the injured area, portable and self-administered, it is superior to other treatment technologies. SAM has achieved widespread use and acceptance among sports medicine professionals and the military because of its effectiveness and ease of use. Multiple studies have confirmed the real-world healing and pain reduction provided by SAM. The survey and panel review data from healthcare providers confirmed the efficacy profile of SAM treatment and expected patient outcomes. A significant 83.9% of the time, SAM was prescribed for healing specific tendon and ligament overuse injuries with demonstrated therapeutic healing outcomes in as little as 1-2 weeks of daily use. Accelerated healing from SAM treatment also directly reduced the use of pain medication for over 50% of treated patients. Patients were receptive or very receptive to SAM treatment. Providers reported a significant 81.2% confidence that SAM enables injured athletes to quickly recover with less downtime, a faster and more sustained return to work, better morale, and relief from pain. The authors concluded that there was consistency between the data presented and the findings. The application of SAM treatment accelerated the rehabilitation process and pain alleviation leading to a faster and more sustained return to work.

## Figures and Tables

**Table 1.** Survey questions provided to ATs for a response.

Survey Questions
Q1. Number of years being an AT?
Q2. What healthcare sector do you work in as an AT?
Q3. What geographical region do you work in as an AT?
Q4. Years of SAM use in clinical practice?
Q5. The average age of patients you treat with SAM?
Q6. How many patients have you treated with SAM?
Q7. Where do you most commonly use SAM treatment on the body?
Q8. When using SAM treatment, typically, how long does it take for your patient to respond to therapy?
Q9. In the last 3-months, how often do you apply SAM with topical drugs mixed into the patch when treating patients?
Q10. The primary reason I use SAM treatment is for?
Q11. How many hours each day is your typical SAM treatment?
Q12. How receptive are your patients to SAM treatment and follow your instructions?
Q13. How has SAM treatment helped your patients reduce the need for oral pain medication?
Q14. As a healthcare provider, what is your expectation/confidence level for your patient’s positive response to SAM treatment after 30 days of regular use?
Q15. Recently published research on SAM treatment with topical diclofenac has demonstrated significant pain, global health, and quality measure improvements for patients. If you have experience with this type of treatment on patients, how would you rate your overall satisfaction?
Q16. From your perspective as a healthcare provider, what is your athlete’s general mental health state when a musculoskeletal or bone injury occurs that prevents participation in sport?
Q17. When using SAM to treat an injury or re-injury, what confidence level when they return to the field/court of play?
Q18. How important is it for your patients that SAM treatment is covered under insurance and health benefits?

**Table 2.** Regional response rate is written the survey.

Survey Participants by Region	

NATA Region	Number of Participants (n=97)

Eastern (District 1)	3
Eastern (District 2)	8
Mid-Atlantic (District 3)	16
Great Lakes (District 4)	9
Mid America (District 5)	4
Southwest (District 6)	8
Rocky Mountain (District 7)	7
Far West (District 8)	10
Southeast (District 9)	21
Northwest (District 10)	5
Other answers	6

**Table 3.** Results from the ATs survey on athletes’ response to sustained acoustic medicine (SAM) following injury.

Survey Result on Patient Receptivity, Medication Use, Sonophoresis, and Confidence in Treatment			
Question for AT in relation to SAM	Less than 25%	50%	75-100%
The percentage of our injured athletes receptive to using SAM to heal.	0	12.6%	87.4% *(p<0.001)
Percent of our athletes who reduce the use of oral pain medications because of SAM.	17.4%	62.8% *(p<0.001)	19.8%
Percent of our athletes who receive sonophoresis of NSAID with SAM therapy.	54.0%	23.0%	23.0%
Percent of confidence in positive healing response to SAM treatment after 30 days of regular use	5.8%	12.8%	81.4% *(p<0.001)

## Data Availability

Not applicable.

## LIST OF ABBREVIATIONS

SAM: Sustain Acoustic Medicine
FDA: Federal Drug Administration
NATA: National Athletic Trainers Association
NSAIDs: Nonsteroidal Anti-inflammatory Drugs
RICES: Rest, Ice, Compression, Elevation, Stabilization
EMS: Electrical Muscle Stimulation
TENS: Transcutaneous Electrical Stimulation
PRP: Platelet-Rich Plasma
ATs: Athletic Trainers
VAS: Visual Analog Scale
NRS: Numerical Rating Scale
GROC: Global Rate of Change
WOMAC: Western Ontario McMaster Osteo-Arthritis Index
